# Cloning and Characterization of Two *MAPK* Genes *UeKpp2* and *UeKpp6* in *Ustilago esculenta*

**DOI:** 10.1007/s00284-018-1483-3

**Published:** 2018-03-28

**Authors:** Yafen Zhang, Qianwen Ge, Qianchao Cao, Haifeng Cui, Peng Hu, Xiaoping Yu, Zihong Ye

**Affiliations:** Zhejiang Provincial Key Laboratory of Biometrology and Inspection & Quarantine, College of Life Sciences, China Jiliang University, Hangzhou, 310018 Zhejiang China

## Abstract

*Ustilago esculenta*, resembling a fungal endophyte in *Zizania latifolia*, inhibits the host plant flowering and induces the host stems to swell and form edible galls. It is well believed that when and how the fungus infects and proliferates in the host plants during the host development is of importance in the edible gall formation. Mitogen-activated protein kinases (MAPKs) have been found to play an important role in sensing environment cues and regulating infection. Two *MAPK* genes *UeKpp2* and *UeKpp6* from *U. esculenta* were cloned and suggested to be involved in the Fus3/Kss1 pathway by a phylogenetic analysis with the neighbor-joining method. Quantitative RT-PCR (qRT-PCR) analyses indicated that expression of *UeKpp2* and *UeKpp6* were induced during mating and infection processes, and their expression patterns displayed differentially under different carbon and nitrogen sources. In addition, subcellular localization of UeKpp2 or UeKpp6 fused with the reporter green fluoresce protein was observed by confocal laser scanning microscope, and yeast two-hybrid assays were carried out. Results showed that both UeKpp2 and UeKpp6 were located in cytoplasm and interacted with UePrf1, indicating their involvement in hyphal growth and host–pathogen regulation. Only UeKpp2 but not UeKpp6 interacted with the upstream MAPK kinase UeFuz7, implying an additional MAPK pathway, in which UeKpp6 involved, existed.

**Electronic supplementary material:**

The online version of this article (10.1007/s00284-018-1483-3) contains supplementary material, which is available to authorized users.

## Introduction

*Ustilago esculenta*, belonging to the Ustilaginaceae of basidiomycetes, resembles an endophytic fungus of *Zizania latifolia*, a perennial aquatic grass widely cultivated in southeastern Asian countries [1]. The *U. esculenta*-infected host plants, commonly called *jiaobai* in China, fail to flower and produce seeds, whereas the plant upper stems swell and form edible galls. In the fields, the majority of swollen galls with white appearance and soft tissues, named white *jiaobai*, are consumed as an aquatic vegetable for its flavor and delicacy [2]. But galls filled with dark-colored teliospores, called grey *jiaobai*, always appear in a harvest season [3]. They are often discarded because they seriously distort the quality of *jiaobai*. Previous studies reported that the strains of *U. esculenta* from white *jiaobai* and grey *jiaobai* were distinct, characterized as mycelia–teliospore (MT) and teliospore (T) strains [4], respectively. It was speculated that the strains of MT and T are different in pathogenicity or teliospore formation [4].

It is generally accepted that mating of two compatible haploid strains to form invasive hyphae is essential for fungal infection in Ustilaginaceae [5]. During mating and hyphal formation, MAPK signaling cascades (consist of MAP kinase kinase kinase (MAPKKK), MAP kinase kinase (MAPKK), and MAP kinase (MAPK) sequentially) play crucial roles in the transduction of extracellular cues and fungal pheromone response [6, 7]. Besides, MAPKs show different functions. For instance, in *Saccharomyces cerevisiae*, Fus3 regulates inter gametophytic mating and Kss1 is primarily responsible for the following invasion [8]. In *Candida albicans*, Cek1 and Cek2 have overlapping functions in the mating progress and filamentous growth [9]. In *Ustilago maydis*, which is closely related to *U. esculenta*, Ubc3/Kpp2, and Kpp6 has been proven to function in mating and plant infection [10, 11]. Fuz7, which functions upstream of Kpp2 and Kpp6 [12], plays an important role in conjugation tube and filament formation, and the maintenance of filamentous growth in *U. maydis* [13]. The pheromone response factor Prf1, which could be phosphorylated by Kpp2 and Kpp6 [14], is critical in mating, filamentous growth, and pathogenicity in *U. maydis* [15]. In addition, Ubc3/Kpp2 could be phosphorylated by activated Fuz7 and then regulate both the prf1-dependent transcriptional response to pheromone and the prf1-independent formation of conjugation tube [12]. Furthermore, the distinct N-terminus of Kpp6 has been proven having an inhibitory function in pathogenicity, in which a PR-motif (proline-rich motif) is located to interact with the SH3 domain of Sho1 [16]. What MAPKK activates Kpp6, however, remains unknown [17, 18].

Recently, the differentiations of MT and T strains were deeply investigated. Growth and pathogenicity defects in MT strains were discovered and a distinct mating response to different culture media and culture time between the MT and T type strains were proved [19]. In this report, in order to discover the different mating response mechanisms that determine pathogenicity difference, we cloned two MAP kinase genes *UeKpp2* and *UeKpp6* from *U. esculenta*, and analyzed their relative expression during mating/infection progresses and under different exogenous stimuli. In addition, we employed a yeast two-hybrid assay to study the interaction between UeKpp2/UeKpp6 and UeFuz7/UePrf1. We also fused *UeKpp2*/*UeKpp6* to the reporter gene *EGFP* to investigate the subcellular localization of UeKpp2 and UeKpp6 during the mating process.

## Materials and Methods

### Strains and Growth Conditions

Compatible haploid *U. esculenta* strains UeMT10 (a3b3 CCTCC AF 2015020)/UeMT46 (a2b2 CCTCC AF 2015021) isolated from white *jiaobai* and UeT14 (a1b1 CCTCC AF 2015016)/UeT55 (a2b2 CCTCC AF 2015015) isolated from grey *jiaobai* were grown at 28 °C on the YEPS (1% yeast extract, 2% peptone, and 2% sucrose) solid medium. The *S. cerevisiae* strain Y2HGold and Y187 (Clontech) were used for two-hybrid interaction studies. The tubular stems of *Z. latifolia* (longjiao 2#) plants were dug up from field and planted into a mixed soil (nutritive soil: vermiculite: perlite 4:4:1) in greenhouse under a 8 h light (~ 800 µmol s^−1^ m^−2^ photons m^−2^ s^−1^ of intensity) and 16 h dark cycle at 15 ± 2 °C with 80% relative humidity until the tubular stems grow to tillering stage. Then change the conditions to a 12 h light and 12 h dark cycle at 25 ± 2 °C with 80% relative humidity. Stem tip samples were obtained from the three important periods, tillering stage (the day before the culture conditions change, ~ 20 days after tubular stems germination), 8-leaf stage (~ 20 days after the culture conditions changed), swollen stage (~ 40 days after the culture conditions changed), respectively.

### Gene Cloning

The genomic DNA was extracted with Ezup Column Fungi Genomic DNA Purification Kit (Sangon Biotech, China). Total RNA was isolated according to the RNAiso Plus’ instructions (Takara, Japan). The cDNAs were synthesized using PrimeScript™ II 1st strand cDNA Synthesis Kit (Takara, Japan). Specific primers (supplementary data 1) were designed according to genome sequencing data. PCR was performed with LA Taq DNA polymerase (Takara, Japan). The objective PCR products were purified with SanPrep Column DNA Gel Extraction Kit (Sangon Biotech, China) and cloned into pMD19-T (Takara, Japan), and then confirmed by sequencing.

### Bioinformatics Analysis

The open reading frame (ORF) of *UeKpp2* and *UeKpp6* were determined using the ORF finder (http://www.ncbi.nlm.nih.gov/gorf/). The conserved motifs and predicted domains were analyzed at NCBI. Multiple alignments of the putative proteins were performed with the DNAMAN 8.0 software. Phylogenetic analysis of MAP kinases from other related fungi was performed with the MEGA 5.0 program using the neighbor-joining method.

### Mating Assays

Haploid strains UeMT10, UeMT46 and UeT14, UeT55 were cultured in the YEPS liquid medium to an OD_600_ of 1.0–1.5, and then collected and resuspended in the YEPS liquid medium to an OD_600_ of 2.0. After compatible haploid strains mixed, 3 µl of cells was dropped to culture on the YEPS solid medium or a prepared medium with indicated compounds (with different carbon or nitrogen sources described below) at 28 °C. Samples were collected at 0, 12, 24, 36, 48, 60 h. After 60 h, mating colonies were observed with microscopy. Media for treatments were prepared with basic medium (BM) and indicated carbon or nitrogen sources. Basic medium was prepared as follows: K_2_HPO_4_ 1 g, MgSO_4_·7H_2_O 0.5 g, FeSO_4_·7H_2_O 0.01 g, KCl 0.5 g, Agar 15 g, adding distillated water to 1000 ml, and autoclaving for sterilization. Nitrogen source (NH_4_NO_3_, methionine, arginine, or urea) was added to BM to make the c (N) = 20 mM/l, filtered by Millipore filters (0.22 µm), with sucrose as carbon source (50 mM/l), being nitrogen sources changed medium. Carbon source (galactose, glucose, fructose, sorbitol, mannitol, lactose, or maltose) was added to BM to make the c (C) = 600 mM/l, filtered by Millipore filters (0.22 µm), with KNO_3_ as nitrogen source (20 mM/l), being carbon sources changed medium. The BM with 20 mM/l KNO_3_ and 50 mM/l sucrose was prepared as blank control (CK).

### Expression Analysis by qRT-PCR

The cDNAs were synthesized using the PrimeScript™ RT reagent Kit with gDNA Eraser (Takara, Japan). qPT-PCR was performed with SYBR Premix EX Taq™ (TliRNaseH Plus) (Takara, Japan) on the ABI 7500 Real-time Detection System (Applied Biosystems, USA) using the gene-specific RT primer pairs (supplementary data 1). *β-Actin* was used as the internal reference for measuring gene expression. The comparative CT (2^−△△CT^) method was used for calculating relative gene expression [20].

### Subcellular Localization of MAPK-GFP Chimeric Proteins

The MAPK-eGFP chimeric fragments were gained by fusion PCR with specific primers (Supplementary data 1) and linked by the PG linker proteins [21]. Then recombinant plasmid pUMa932-UeKpp2/UeKpp6-eGFP was constructed and cut to linear segments by QuickCut™ NdeI (Takara, Japan) for next transformation into UeT14 haploid strain using an efficient genetic manipulation protocol reported before [22]. GFP fluorescence was observed using a confocal laser scanning microscope (Leica Microsystems, USA).

### Yeast Two-Hybrid Assay

Yeast two-hybrid assay was performed by the Matchmaker™ Gold Yeast Two-Hybrid System (Clontech). The coding regions of *UeKpp2* and *UeKpp6* were cloned into the bait vector pGBKT7 and transformed into yeast strain Y2HGold. The coding regions of *UeFuz7* and *UePrf1* were cloned into the prey vector pGADT7 and transformed into yeast strain Y187. Subsequently Y2HGold and Y187 carrying recombinant plasmids were mixed and plated on the SD-Leu-Trp medium for initial selection. Transformants were selected randomly and streaked on the SD/-Leu/-Trp/-His/-Ade/X-α-Gal medium for a protein–protein interaction test.

### Statistical Analysis

All experiments were performed in triplicates and data were shown as mean ± SD from the three independent experiments. All data were analyzed according to the Duncan’s method. The probability values of *P* < 0.05 were considered as significant.

## Results

### Identification of Two MAP Kinases *UeKpp2* and *UeKpp6* in *U. esculenta*

BlastP searches against the genomic database of *U. esculenta* (JTLW00000000) were performed using the Kpp2 and Kpp6 protein sequences of *U. maydis* as queries. Two predicted loci (g262 with 96% identity to Kpp2, and g4166 with 83% identity to Kpp6) were obtained. The full-length cDNAs of the two predicted genes were cloned and confirmed by sequencing. One encoding a protein of 354 amino acids (Supplementary data 2A) with a high identity to Kpp2, was named *UeKpp2* (Accession Number: KU855052). UeKpp2 has an estimated molecular mass of 90.56 kDa and a predicted pI of 5.00. Another gene coding for 578 amino acids (Supplementary data 2B) with a high identity to Kpp6 was named *UeKpp6* (accession number: KU855053). UeKpp6 possesses an estimated molecular mass of 148.52 kDa with a predicted pI of 4.88. There is no intron in *UeKpp2* and *UeKpp6*, and their encoding amino acid sequences were identical in the MT and T type strains.

Furthermore, the STKc_ERK1_2_like family region and conserved TEY dual phosphorylation sites, which have been shown to be phosphorylated upon stimulation through its MAPK kinases [23], were found in UeKpp2 and UeKpp6 (Supplementary data 2). Interestingly, being similar to what was found in *U. maydis* [11], an additional N-terminal domain with a perfect match box to the Prf1 consensus binding site (ACAAAGGGA) exists in both proteins. An alanine linker encoded by a trinucleotide repeat domain (GCT)n found in Kpp6 [11], does not exist in UeKpp6.

**Fig. 1:**
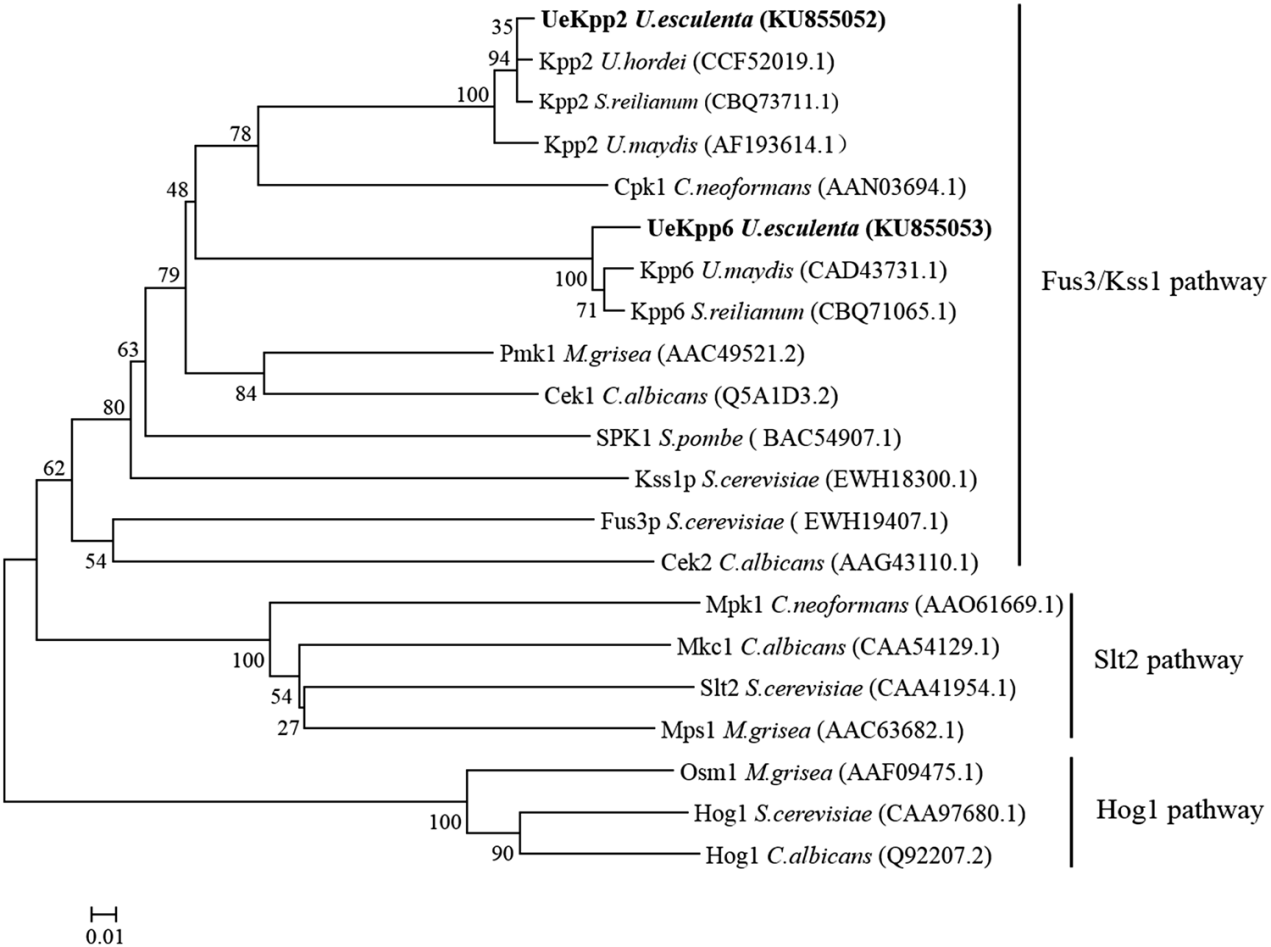
Phylogenetic tree constructed from the MAP kinase protein sequences of several fungi. Amino acid sequences were used and analyzed by MEGA 5.0 with the neighbor-joining method. Numbers on the branches represent bootstrap support for 1000 replicates. The related MAPK proteins used were: *Ustilago esculenta* UeKpp2(KU855052) and UeKpp6 (KU855053), marked in bold; *Ustilago hordei* Kpp2 (CCF52019.1); *Sporisorium reilianum* Kpp2 (CBQ73711.1) and Kpp6 (CBQ71065.1); *Ustilago maydis* Kpp2 (AF193614.1) and Kpp6 (CAD43731.1); *Cryptococcus neoformans* Cpk1 (AAN03694.1) and Mpk1 (AAO61669.1); *Magnaporthe grisea* Pmk1 (AAC49521.2), Mps1 (AAC63682.1) and Osm1 (AAF09475.1); *Candida albicans* Cek1 (Q5A1D3.2), Cek2 (AAG43110.1), Mkc1 (CAA54129.1) and Hog1 (Q92207.2); *Schizosaccharomyces pombe* SPK1 (BAC54907.1); *Saccharomyces cerevisiae* Kss1p (EWH18300.1), Fus3p (EWH19407.1), Slt2 (CAA41954.1) and Hog1 (CAA97680.1)

Sequence alignment and phylogenetic tree analysis revealed that UeKpp2 showed more than 96% of identity to Kpp2 from smut fungi [10, 24], 55–75% of identity to other homologs in pathogenic fungi, such as Pmk1 in *M. grisea* [25], Fus3p [26], and Kss1p [8] in *S. cerevisiae*, Cek1 in *C. albicans* [27], whereas UeKpp6 showed a lower identity, nearly 80% to Kpp6 from smut fungi, 35% to other MAP kinases. The two MAP kinases were both clustered into Fus3/Kss1 pathway (Fig. 1), and showed high levels of similarity to smut fungi such as *U. maydis, Ustilago hordei, Sporisorium reilianum* (Fig. 1 and Supplementary data 3), implying that they might be involved in mating response and pathogenicity [7].

### The Expression of *UeKpp2* and *UeKpp6* was Induced During Mating and Infection Progresses

**Fig. 2:**
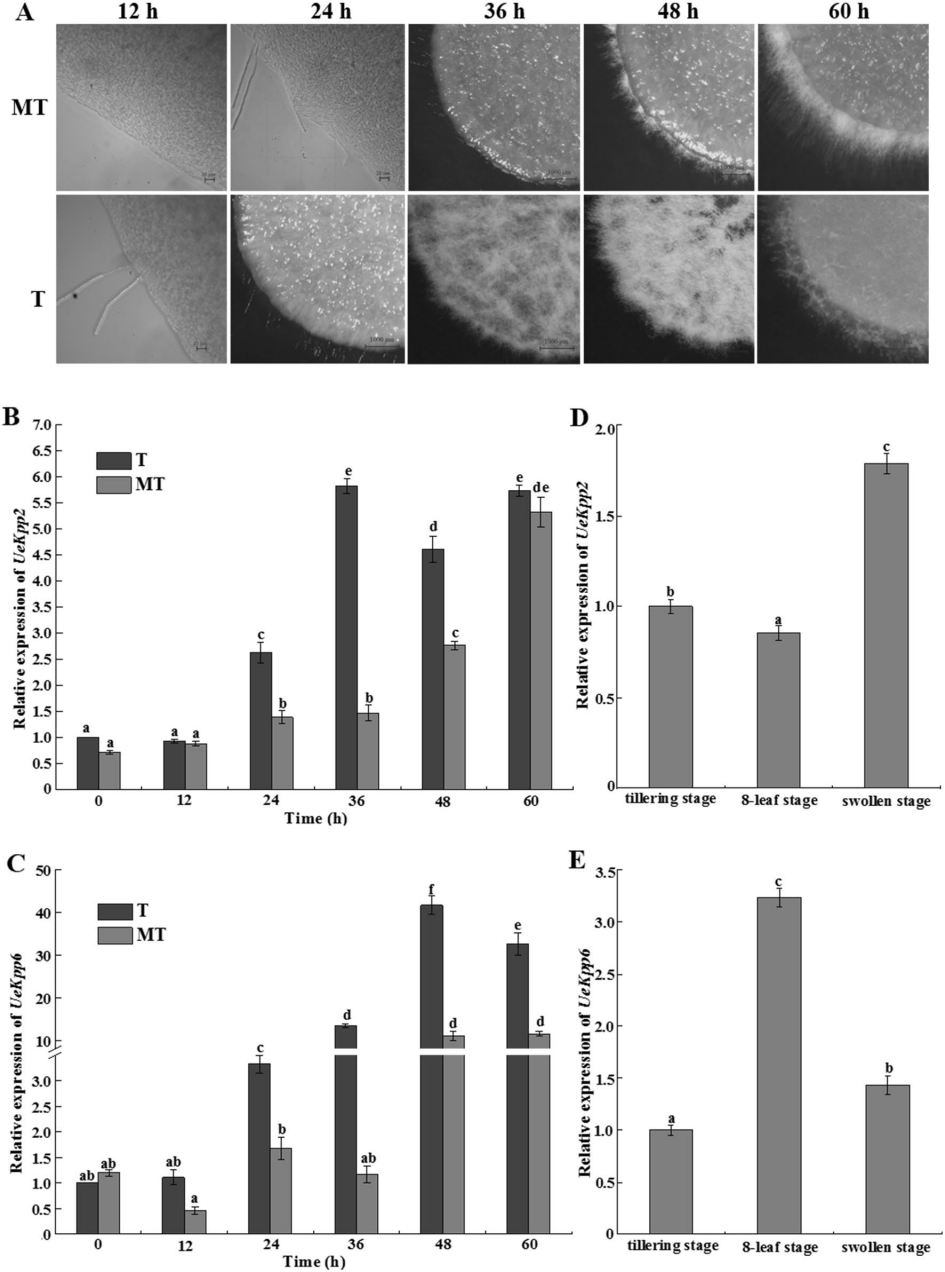
The relative expression of *UeKpp2* and *UeKpp6* during mating and infection progress. **a** Mating phenotype of MT and T type strains at different time with 12 h intervals. Earlier photos were taken by an ordinary microscope with the bars of 20 µm; Other images were taken by a stereomicroscope with the bars of 1000 µm; Relative expression of *UeKpp2* (**b**) and *UeKpp6* (**c**) were analyzed during different mating time; Relative expression of *UeKpp2* (**d**) and *UeKpp6* (**e**) during infection progress were also analyzed. *β-Actin* was used as an internal control. Data were reported as means ± SD of three repeated samples. All data were subjected to statistical analyses according to the Duncan’s method. The probability values of *P* < 0.05 were considered as significant and reported with the letter notation

To explore the possible roles of UeKpp2 and UeKpp6 in mating response and pathogenicity, expression patterns of *UeKpp2* and *UeKpp6* during mating and infection progresses were analyzed. In the T type strains, hyphae were observed at 12 h and the majority of white and fuzzy filaments appeared at 36 h, while the MT type strains showed 12 h later in hyphae formation and weaker hyphal growth (Fig. 2a). Meanwhile, temporal expression results showed that during mating process, *UeKpp2* and *UeKpp6* were up-regulated in hyphal growth phase than in cells budding phase, especially after white and fuzzy filaments formation, and showed a distinctly higher expression in the *T* type strains after the hyphal formation when compared to the MT strains, indicating their involvement in hyphal growth. In addition, relative expression of *UeKpp2* was increasing and peaked at 36 h in the T type strains but 60 h in the MT type strains (Fig. 2b), while *UeKpp6* increased to the highest expression level at 48 h (Fig. 2c), indicating an unknown and distinct role of *UeKpp2* and *UeKpp6* in the MT and T type strains.

To analyze potential roles of *UeKpp2* and *UeKpp6* in the fungal infection progress during host growth and development, three related periods of the host plant were selected and the relative expression patterns of *UeKpp2* and *UeKpp6* in these three periods were determined. The first period is the tillering stage when the fungi are trying to go into the different seedlings. The second is the 8-leaf stage that is supposed to the key period for the fungi infection and spread. The third is the swollen stage when the fungi are widely distributed throughout the swollen tissues. A remarkable increase of *UeKpp6* in the second and key period (Fig. 2e) and high expression of *UeKpp2* in the swollen stage were observed (Fig. 2d), indicating their involvement in the interaction between *U. esculenta* and *Z. latifolia*, especially for *UeKpp6*.

### *UeKpp2* and *UeKpp6* Showed Different Expression Pattern in Response to Distinct Nitrogen or Carbon Source Treatment

**Fig. 3:**
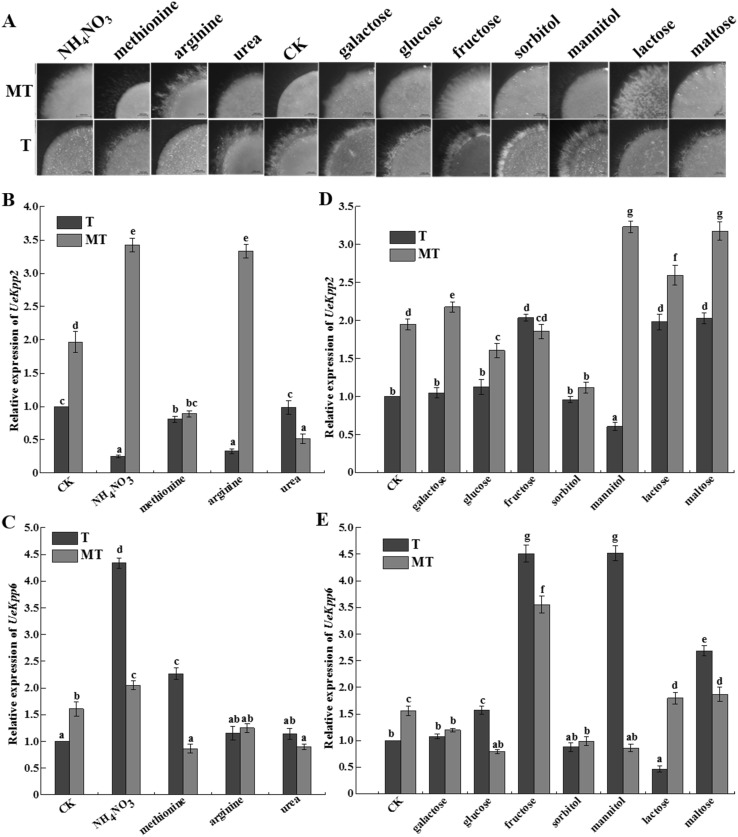
The expression patterns of *UeKpp2* and *UeKpp6* under distinct nitrogen or carbon source treatments. **a** Mating phenotype of MT and T type strains on a solid culture medium with different nitrogen and carbon sources. Images were taken after 3 days by a stereomicroscope. Bars indicate 1000 µm. Relative expression of *UeKpp2* (**b**) and *UeKpp6* (**c**) in distinct nitrogen sources were analyzed; Also, Relative expression of *UeKpp2* (**d**) and *UeKpp6* (**e**) were analyzed in distinct carbon source treatment. The relative expression in the control medium with sucrose (50 mM) and KNO_3_ (20 mM) was taken as one fold relative expression sample. *β-Actin* was used as an internal control. Data were reported as means ± SD of three repeated samples. All data were subjected to statistical analyses according to the Duncan’s method. The probability values of *P* < 0.05 were considered as significant and reported with the letter notation

To find out whether *UeKpp2* and *UeKpp6* in *U. esculenta* respond to external stimuli or not, especially during starvation of nutrition [7], distinct nitrogen and carbon source treatment assays were carried out. The results showed that methionine, arginine obviously promoted the hyphal growth of the MT type strains, while NH_4_NO_3_ and arginine inhibited the hyphal growth of the T type strain (Fig. 3a). The relative expression of *UeKpp2* was up-regulated in the MT strains and down-regulated in the T strains significantly, under the stimulus of NH_4_NO_3_ and arginine (Fig. 3b), indicating a consistent association of *UeKpp2* with hyphal growth. However, it showed no difference of expression of *UeKpp6* in the T and MT type strains under various nitrogen source treatments except the stimulus of NH_4_NO_3_ which gave more than threefolds higher expression of *UeKpp6* in the T type strains (Fig. 3c).

In addition, the MT strains showed a defect in mating under a variety of carbon sources except mannitol while the T strains displayed a normal growth (Fig. 3a). As a whole, the expression of *UeKpp2* in the MT type strains was more obviously higher than that in the T type (Fig. 3d). When compared to the control carbon source, the relative expression of *UeKpp2* in the MT strains was up-regulated under mannitol, lactose, and maltose treatment but down-regulated under sorbitol treatment, while fructose and disaccharide promoted expression of *UeKpp2* in the T strains (Fig. 3d). Also, fructose, mannitol, and maltose could promote expression of *UeKpp6* significantly, while lactose could suppress expression of *UeKpp6* in the T strains (Fig. 3e).

### UeKpp2 and UeKpp6 Were Localized in Cytoplasm and Interacted with UePrf1 in Vitro

**Fig. 4:**
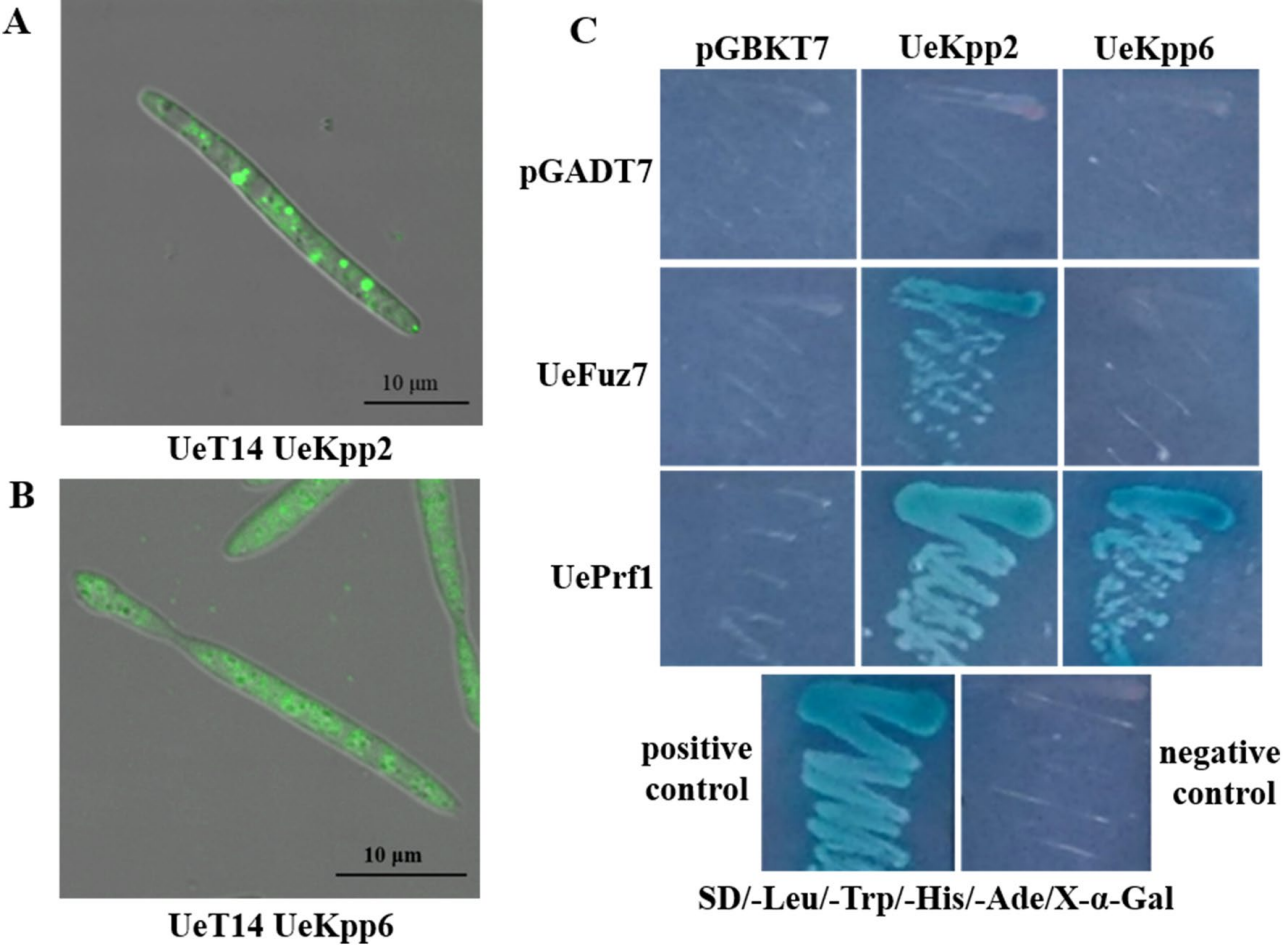
Subcellular location and protein interaction of UeKpp2 and UeKpp6. Subcellular location of UeKpp2 (**a**) and UeKpp6 (**b**) were studied in *U. esculenta*. Images were taken by a confocal laser scanning microscope. Bars indicate 10 µm. **c** The interaction between the two MAP kinases and UeFuz7/UePrf1 from *U. esculenta*. pGBK7 or its derivatives (as indicated on top) were transformated into Y2HGold. pGADT7 and its derivatives (as indicated on left) were transformated into Y187. Y2HGold [pGBKT7-53] and Y187 [pGADT7-T] were used as a positive control. Y2HGold [pGBKT7-Lam] and Y187 [pGADT7-T] were used as a negative control

In order to reveal the functional mechanism of UeKpp2 and UeKpp6 in *U. esculenta*, over-expression of pUMa932-UeKpp2/UeKpp6-GFP were conducted in UeT14 haploid strain to analyze subcellular localization of UeKpp2 and UeKpp6 under mating conditions. As shown by fluorescence microscopy, both UeKpp2 and UeKpp6 were localized in the cytoplasm, but UeKpp2 showed an aggregated distribution pattern (Fig. 4a, b). In addition, the yeast two-hybrid assays were carried out and results showed that UeKpp2 and UeKpp6 were able to interact with UePrf1 (ALS87609.1) (Fig. 4c), which is in agreement to the function of MAP kinases in *U. maydis* [12]. However, only UeKpp2 interacted with UeFuz7 (KX369240) (Fig. 4c).

## Discussion

MAPK signaling pathways play essential roles in many aspects of fungal development, such as mating, appressorium formation, and pathogenesis or virulence [7], especially the Fuz3/Kss1 pathway, a common pathway in many fungi, such as, *U. maydis, M. grisea, and C. albicans*. In this study, two *MAPK* genes *UeKpp2* and *UeKpp6* from *U. esculenta* were cloned and their putative functions were proposed in the Fuz3/Kss1 pathway because they possess a TEY motif in the activation loop of subdomain VIII (Fig. 1) which is a common character for the Fus3/Kss1 family [23] (Supplementary data 3). The *UeKpp2* expression profile matched well with the hyphal growth in the T and MT type strains (Fig. 2a, b) and was up-regulated at the later stage during infection (Fig. 2d), while *UeKpp6* showed a highly up-regulated expression at the later phase of hyphal growth in T and MT type strains (Fig. 2c) and at an earlier stage in the infection progress (Fig. 2e). The result indicated that, similar to *Kpp2* and *Kpp6* in *U. maydis* [10–12], *UeKpp2* might be more related to transmitting pheromone signals, while *UeKpp6* might play a more important role in the interaction between *U. esculenta* and *Z. latifolia*.

In response to the environment cues such as distinct nitrogen and carbon sources, *UeKpp2* and *UeKpp6* showed more sensitive to nitrogen sources than carbon sources and their responsive patterns were different (Fig. 3). In more details, the expression pattern of *UeKpp2* to the arginine and NH_4_NO_3_ treatments indicated its role in mating in vitro, because of its up-regulation in the MT type strains with promoted hyphal growth and down-regulation in the T type strains with inhibited hyphal growth (Fig. 3a, b). But *UeKpp6* might have an undiscovered role in the nitrogen response because in the methionine and NH_4_NO_3_ treatments, the expression of *UeKpp6* was up-regulated in the T type strains with inhibited hyphal growth but down-regulated in the MT type strains with promoted hyphal growth (Fig. 3a, c), which is opposite to the predicted mating regulation roles. We have also found that both UeKpp2 and UeKpp6 were located in cytoplasm and interacted with UePrf1 (Fig. 4), which is similar to the case in *U. maydis* and have been proved to be important in hyphal growth and host–pathogen regulation [12], providing further evidence that UeKpp2 and UeKpp6 participate in hyphal growth and stem swollen induction. The story that only UeKpp2 but not UeKpp6 could interact with UeFuz7 (Fig. 4c), a homolog to Fuz7 in *U. maydis* [12], indicates that UeKpp6 may be involved in other MAPK cascades in response to environment cues. The detailed function of UeKpp6 in other MAPK cascades needs to be investigated. Therefore, the studies of *U. esculenta* are of importance, particularly in the sense of helping discover distinct MAPK cascades in the interaction system of *U. esculenta* and *Z. latifolia*, since *Z. latifolia* was the only host to date in forming edible vegetable because of *U. esculenta* infection [2].

## Electronic supplementary material

Below is the link to the electronic supplementary material.

Supplementary material 1 (DOCX 1197 KB)
