# Supplementary material for: Cloning and Characterization of Two MAPK Genes UeKpp2 and UeKpp6 in Ustilago esculenta
Source: Curr Microbiol. 2018 Mar 28;75(8):1016–24. doi: 10.1007/s00284-018-1483-3 (PMC6018589; doi:10.1007/s00284-018-1483-3)
Supplement: Supplementary file 1 — Supplementary material 1 (DOCX 1197 KB) [file 284_2018_1483_MOESM1_ESM.docx]

| Primer | Sequence(5’→3’) | Purpose |
| --- | --- | --- |
| UeKpp2-gF | GCTTTGAACCGTTTGTGAGC | clone |
| UeKpp2-gR | TATACAGCGAAGTCGCCAAC | clone |
| UeKpp6-gF | TTGTTTGGTCGCGCTGCAGG | clone |
| UeKpp6-gR | TGAAGAGCTTCAAGACGGAG | clone |
| UeKpp2-cF | ATGGCGCACGCACATGGAC | clone |
| UeKpp2-cR | TCAACGCATGATCTCATTGTAG | clone |
| UeKpp6-cF | ATGGCCTCCACCGACCACG | clone |
| UeKpp6-cR | TCAGCGAAGAAGGGGCTGG | clone |
| UeKpp2-F | CATGCCATGGCGCACGCACATGGACAG | clone |
| UeKpp2-R | CACCATACCAGGACCAGGACGCATGATCTCATTGTAGA | clone |
| UeKpp6-F | CATGCCATGGCCTCCACCGACCACGC | clone |
| UeKpp6-R | TCACCATACCAGGACCAGGGCGAAGAAGGGGCTGGAACT | clone |
| eGFP-F | CCTGGTCCTGGTATGGTGAGCAAGGGCGAGGAG | clone |
| eGFP-R | ATAAGAATGCGGCCGCTCACTTGTACAGCTCGTCCATG | clone |
| UeKpp2-qF | CACCTTGGAAATCCTGGGCAC | qPCR |
| UeKpp2-qR | GACGGCGAGAGGATTAGCGTT | qPCR |
| UeKpp6-qF | GCATACTTGCCGAAATGCTCAC | qPCR |
| UeKpp6-qR | CGGCTGTAGATGTTGTGGAACT | qPCR |
| Actin-qF | CAATGGTTCGGGAATGTGC | qPCR |
| Actin-qR | GGGATACTTGAGCGTGAGGA | qPCR |

**Supplementary data 1 Primers used in this study**

**
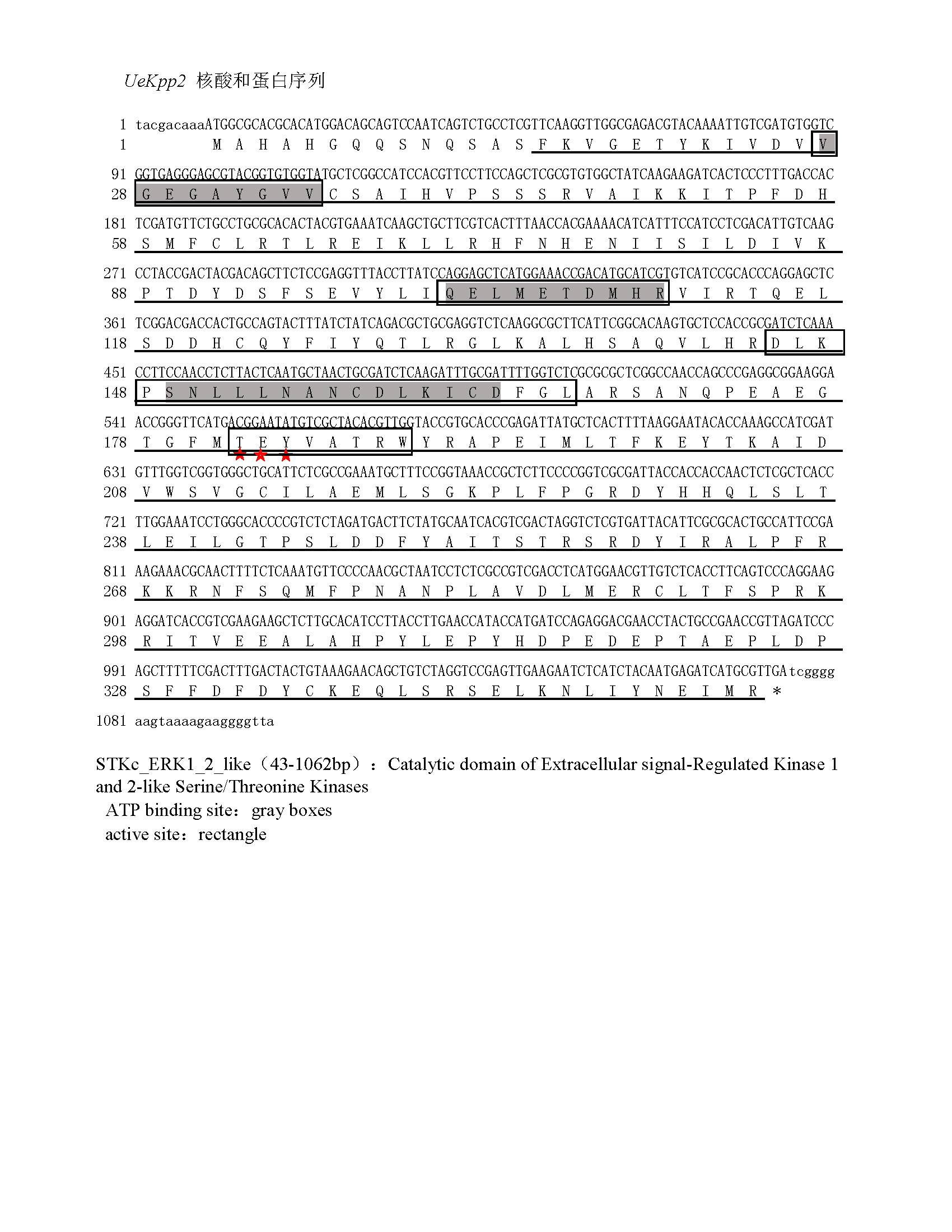
**

A

**
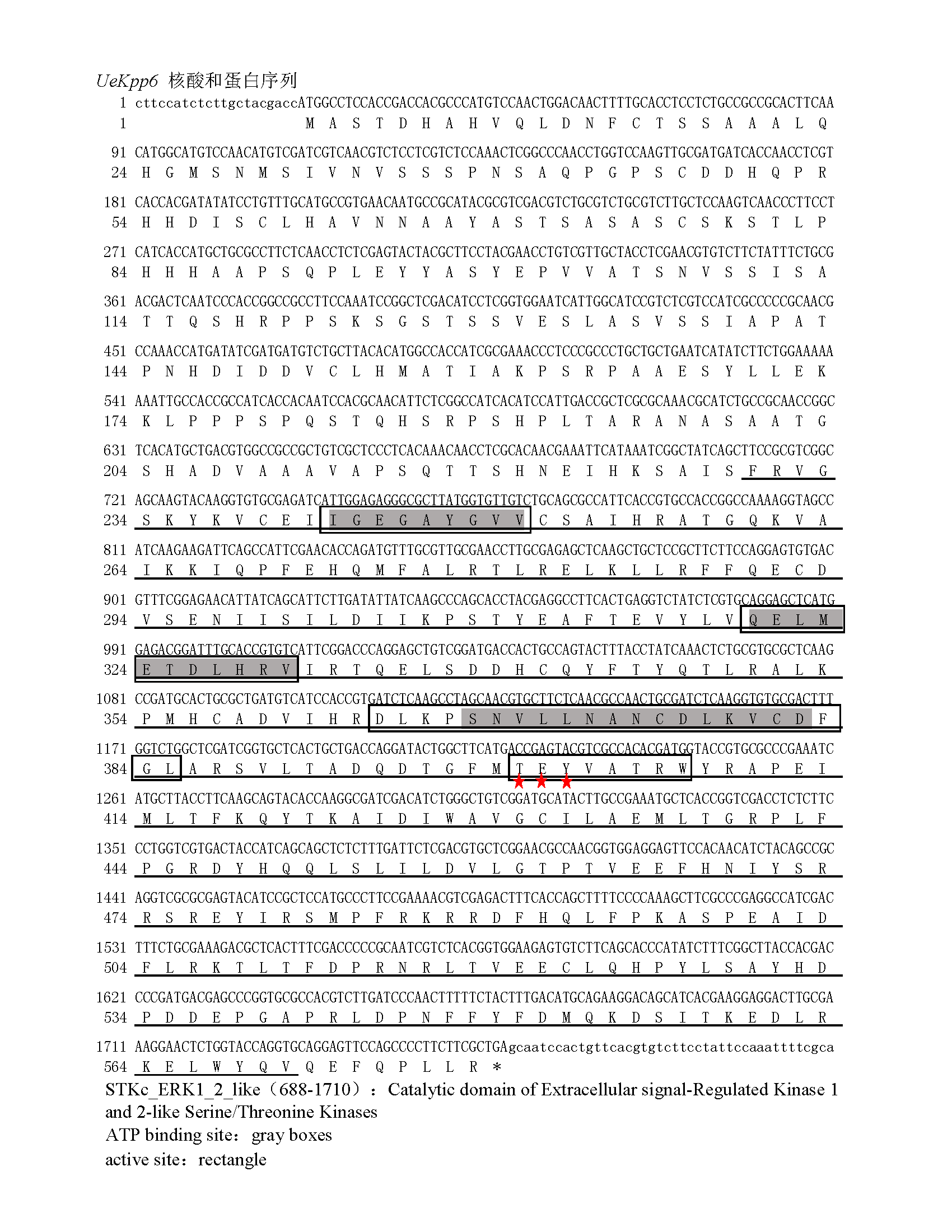
**

B

**Supplementary data 2** **Nucleotide sequences of the two MAPK genes and their deduced amino acid sequences.** A, Nucleotide sequences of *UeKpp2* and its deduced amino acid sequences. B, Nucleotide sequences of *UeKpp6* and its deduced amino acid sequences. The predicted ORF of cDNA are shown by the single–letter amino acid codes below. The STKc_ERK1_2_like family amino acid sequences are marked with *single underline*. The ATP binding sites are represented by the *gray boxes*. *Rectangles* indicate the active sites of the two MAP kinases. Conserved TEY dual phosphorylation sites are shown by *pentagrammes*.

**
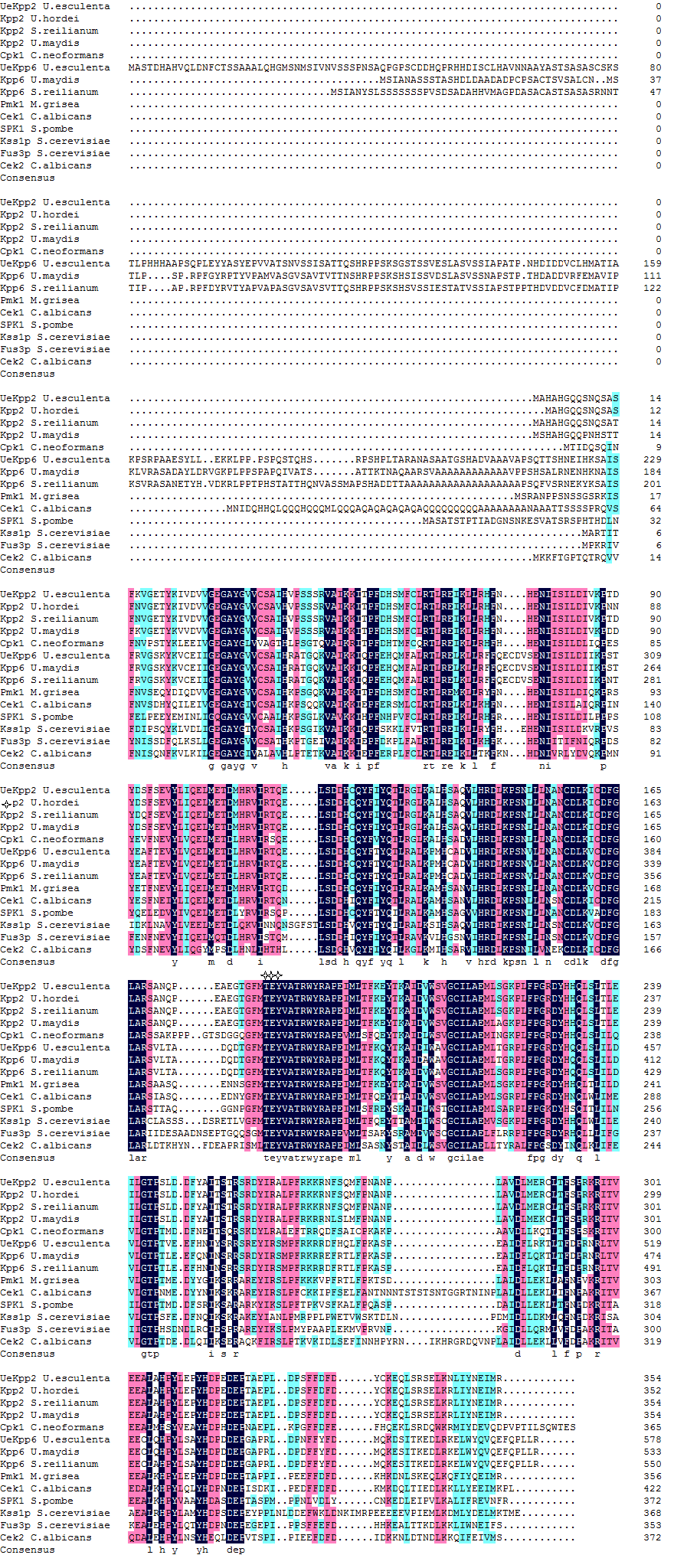
**

# Supplementary data 3 Multiple alignment showing homology of UeKpp2 and UeKpp6 to other MAP kinases from fungi. Conserved residues are shaded by different colour and shurikens denote the invariant TXY motif of MAP kinases. GenBank accession numbers for the displayed proteins are as follows: *Ustilago esculenta* UeKpp2(KU855052) and UeKpp6 (KU855053); *Ustilago hordei* Kpp2 (CCF52019.1); *Sporisorium reilianum* Kpp2 (CBQ73711.1) and Kpp6 (CBQ71065.1); *Ustilago maydis* Kpp2 (AF193614.1) and Kpp6 (CAD43731.1); *Cryptococcus neoformans* Cpk1 (AAN03694.1); *Magnaporthe grisea* Pmk1 (AAC49521.2); *Candida albicans* Cek1 (Q5A1D3.2) , Cek2 (AAG43110.1);*Schizosaccharomyces pombe* SPK1 (BAC54907.1); *Saccharomyces cerevisiae* Kss1p (EWH18300.1), Fus3p (EWH19407.1).
